# 111 Design and implementation of a comprehensive stewardship intervention for a Multiplex Respiratory Panel

**DOI:** 10.1017/ash.2026.10528

**Published:** 2026-06-23

**Authors:** Hamda Memon, Rowena Jimenez, Madeline murphy, Sarah Siemion, Kenneth Paulish

**Affiliations:** 1 Geisinger; 2 Geisinger Wyoming Valley Medical Center

## Abstract

**Background:** Overuse of antibiotics in hospitals leads to resistance to antibiotics, increased consumption of healthcare resources, and adverse drug events. Antibiotic Time-Outs (ATOs) are a recommended antimicrobial stewardship strategy that encourages regular reassessment of antimicrobial therapy that includes indication, spectrum, route, and duration after antibiotic initiation. We evaluated the effect of a hospitalist-led weekly ATO on antibiotic utilization and patient safety outcomes on a high-utilization medical unit. Methods This quality improvement project was conducted on an adult inpatient medical unit with historically high antibiotic use. Baseline antibiotic utilization data were collected from January to March 2025, followed by implementation of weekly hospitalist-led ATO rounds from April-June 2025 with a post-intervention observation phase planned for sustainability assessment. During the ATO rounds, all inpatients receiving systemic antibiotics (excluding those on Infectious Diseases service) were reviewed for indication, microbiology, clinical status, route, and duration. Stewardship recommendations included discontinuation, de-escalation, intravenous-to-oral (IV-to-PO) conversion, and duration optimization. Antibiotic utilization was measured as days of therapy (DOT) per 1,000 patient-days for five targeted agents: ceftriaxone, cefepime, piperacillin-tazobactam, ampicillin-sulbactam, and amoxicillin-clavulanate. Process measures included number and acceptance of stewardship recommendations within 24–48 hours. Clinical outcomes included mortality, length of stay (LOS), and 30-day readmissions for pneumonia, urinary tract infection, cellulitis, sepsis, and diabetic foot infection DRGs. Results Across three months of ATO implementation, 34 patients were reviewed, and 30 stewardship recommendations were issued with 24 recommendations (80%) accepted within 24-48 hours. The most frequent interventions were antibiotic discontinuation, de-escalation of spectrum, and intravenous-to-oral conversion. Compared with the pre-ATO period, the combined DOT for the five targeted antibiotics declined during the ATO phase and was sustained post-intervention, corresponding to an overall ~8% reduction in broad-spectrum IV antibiotic exposure across the study period. Importantly, this reduction was not associated with any signal of harm: mortality, LOS, and 30-day readmission rates for sepsis, pneumonia, UTI, cellulitis, and diabetic foot infections remained stable or improved during both the intervention and post-intervention phases. Conclusion Implementation of a hospitalist-led weekly Antibiotic Time-Out resulted in high acceptance of stewardship recommendations and produced a sustained reduction in broad-spectrum antibiotic exposure without adverse effects on mortality, readmissions, or length of stay. This low-resource, physician-driven intervention represents a scalable and effective approach to improving antimicrobial use and patient safety on high-utilization inpatient units.

